# A Web-Based Health Application to Translate Nutrition Therapy for Cardiovascular Risk Reduction in Primary Care (PortfolioDiet.app): Quality Improvement and Usability Testing Study

**DOI:** 10.2196/34704

**Published:** 2022-04-21

**Authors:** Meaghan E Kavanagh, Laura Chiavaroli, Andrea J Glenn, Genevieve Heijmans, Shannan M Grant, Chi-Ming Chow, Robert G Josse, Vasanti S Malik, William Watson, Aisha Lofters, Candice Holmes, Julia Rackal, Kristie Srichaikul, Diana Sherifali, Erna Snelgrove-Clarke, Jacob A Udell, Peter Juni, Gillian L Booth, Michael E Farkouh, Lawrence A Leiter, Cyril W C Kendall, David J A Jenkins, John L Sievenpiper

**Affiliations:** 1 Department of Nutritional Sciences Temerty Faculty of Medicine University of Toronto Toronto, ON Canada; 2 Clinical Nutrition and Risk Factor Modification Center St. Michael’s Hospital Unity Health Toronto Toronto, ON Canada; 3 Toronto 3D Knowledge Synthesis and Clinical Trials Unit St. Michael’s Hospital Unity Health Toronto Toronto, ON Canada; 4 Department of Nutrition Harvard T.H. Chan School of Public Health Boston, MA United States; 5 Department of Applied Human Nutrition Mount Saint Vincent University Halifax, NS Canada; 6 Departments of Pediatrics and Obstetrics and Gynaecology IWK Health Halifax, NS Canada; 7 Department of Obstetrics and Gynaecology Faulty of Medicine Dalhousie University Halifax, NS Canada; 8 Division of Cardiology St. Michael’s Hospital Unity Health Toronto Toronto, ON Canada; 9 Li Ka Shing Knowledge Institute St. Michael’s Hospital Unity Health Toronto Toronto, ON Canada; 10 Division of Endocrinology and Metabolism St. Michael’s Hospital Unity Health Toronto Toronto, ON Canada; 11 Department of Family and Community Medicine St. Michael’s Hospital Unity Health Toronto Toronto, ON Canada; 12 Department of Family and Community Medicine Temerty Faculty of Medicine University of Toronto Toronto, ON Canada; 13 Family Practice Health Centre Women’s College Hospital Toronto, ON Canada; 14 Peter Gilgan Centre for Women’s Cancers Women’s College Hospital Toronto, ON Canada; 15 School of Nursing Faculty of Health Sciences McMaster University Hamilton, ON Canada; 16 School of Nursing Faculty of Health Sciences Queen's University Kingston, ON Canada; 17 Women’s College Research Institute and Cardiovascular Division Department of Medicine Women’s College Hospital, University of Toronto Toronto, ON Canada; 18 Applied Health Research Centre Li Ka Shing Knowledge Institute of St. Michael's Hospital Department of Medicine, University of Toronto Toronto, ON Canada; 19 Institute for Health Policy, Management, and Evaluation Dalla Lana School of Public Health University of Toronto Toronto, ON Canada; 20 ICES Toronto, ON Canada; 21 Department of Medicine Temerty Faculty of Medicine University of Toronto Toronto, ON Canada; 22 Peter Munk Cardiac Centre and the Heart and Stroke Richard Lewar Centre University of Toronto Toronto, ON Canada; 23 MAP Centre for Urban Health Solutions Li Ka Shing Knowledge Institute St. Michael's Hospital Toronto, ON Canada; 24 College of Pharmacy and Nutrition University of Saskatchewan Saskatoon, SK Canada

**Keywords:** portfolio diet, dietary portfolio, nutrition therapy, dietary application, eHealth, usability testing, quality improvement, mobile phone

## Abstract

**Background:**

The Portfolio Diet, or Dietary Portfolio, is a therapeutic dietary pattern that combines cholesterol-lowering foods to manage dyslipidemia for the prevention of cardiovascular disease. To translate the Portfolio Diet for primary care, we developed the PortfolioDiet.app as a patient and physician educational and engagement tool for PCs and smartphones. The PortfolioDiet.app is currently being used as an add-on therapy to the standard of care (usual care) for the prevention of cardiovascular disease in primary care. To enhance the adoption of this tool, it is important to ensure that the PortfolioDiet.app meets the needs of its target end users.

**Objective:**

The main objective of this project is to undertake user testing to inform modifications to the PortfolioDiet.app as part of ongoing engagement in quality improvement (QI).

**Methods:**

We undertook a 2-phase QI project from February 2021 to September 2021. We recruited users by convenience sampling. Users included patients, family physicians, and dietitians, as well as nutrition and medical students. For both phases, users were asked to use the PortfolioDiet.app daily for 7 days. In phase 1, a mixed-form questionnaire was administered to evaluate the users’ perceived acceptability, knowledge acquisition, and engagement with the PortfolioDiet.app. The questionnaire collected both quantitative and qualitative data, including 2 open-ended questions. The responses were used to inform modifications to the PortfolioDiet.app. In phase 2, the System Usability Scale was used to assess the usability of the updated PortfolioDiet.app, with a score higher than 70 being considered acceptable.

**Results:**

A total of 30 and 19 users were recruited for phase 1 and phase 2, respectively. In phase 1, the PortfolioDiet.app increased users’ perceived knowledge of the Portfolio Diet and influenced their perceived food choices. Limitations identified by users included challenges navigating to resources and profile settings, limited information on plant sterols, inaccuracies in points, timed-logout frustration, request for step-by-step pop-up windows, and request for a mobile app version; when looking at positive feedback, the recipe section was the most commonly praised feature. Between the project phases, 6 modifications were made to the PortfolioDiet.app to incorporate and address user feedback. At phase 2, the average System Usability Scale score was 85.39 (SD 11.47), with 100 being the best possible.

**Conclusions:**

By undertaking user testing of the PortfolioDiet.app, its limitations and strengths were able to be identified, informing modifications to the application, which resulted in a clinical tool that better meets users’ needs. The PortfolioDiet.app educates users on the Portfolio Diet and is considered acceptable by users. Although further refinements to the PortfolioDiet.app will continue to be made before its evaluation in a clinical trial, the result of this QI project is an improved clinical tool.

## Introduction

### Background

The Portfolio Diet, or Dietary Portfolio, is a plant-based dietary pattern of cholesterol-lowering foods that has demonstrated *drug-like* reductions in low-density lipoprotein cholesterol (LDL-C) and other cardiovascular risk factors [1,2]. In a metabolically controlled study, the Portfolio Diet was shown to result in the same LDL-C reduction (approximately 30%) as lovastatin therapy, the first statin to be widely used [1]. In a recent systematic review and meta-analysis, these *drug-like* reductions in LDL-C were confirmed and further benefits were also found on other aspects of the lipid profile (non–high-density lipoprotein cholesterol, apolipoprotein B, and triglycerides), blood pressure, inflammatory markers, and estimated 10-year Framingham risk score compared with a National Cholesterol Education Program Step 2 diet alone [3]. This evidence has led to the recognition of the Portfolio Diet as a therapy for cardiovascular disease management from major international clinical practice guidelines, including the Canadian Cardiovascular Society [4,5], Diabetes Canada [6], Obesity Canada [7], Canadian Cardiovascular Harmonized National Guidelines Endeavour [8], Heart UK [9], European Atherosclerosis Society [10], and the American College of Cardiology and American Heart Association guidelines [11]. Although the Portfolio Diet is recognized by clinical practice guidelines as a preventive nutrition therapy for cardiovascular disease, implementation in clinical practice is limited. Traditionally, nutrition therapy involves multiple face-to-face sessions over an extended length of time with trained personnel. However, many health care providers cite a lack of education, educational materials, and time to counsel their patients on nutrition [12,13]. Advancements in technology may be able to circumvent these issues and expand access to nutrition therapies for patients.

Several studies have shown that health apps can promote positive behavior change and improve related health outcomes. Block et al [14] found that a fully automated intervention targeting nutritional and physical activity behaviors in individuals with prediabetes improved glycemic control and Framingham diabetes risk score over 6 months compared with the waitlist control. In a meta-analysis of 47 randomized controlled trials, Beishuizen et al [15] found that web-based interventions in primary care settings improved risk factors for cardiovascular disease compared with standard of care alone. Thus, health apps can provide an alternative and complementary approach to delivery of preventive nutrition therapy within the limits of primary care, where the shift to remote care during the COVID-19 pandemic has further highlighted the need for evidence-based health apps [16].

Therefore, to translate the current clinical practice guidelines for nutrition therapy for dyslipidemia, we developed a web-based application, the PortfolioDiet.app. The application was developed by an interdisciplinary team of clinical nutrition experts, registered dietitians, cardiologists, and software architects, as well as patient, physician, and dietitian advisory committees. The collaboration with knowledge users throughout the development and testing process is the central premise of the integrated knowledge translation (iKT) approach [17]. The PortfolioDiet.app is currently being used as an optional add-on therapy to the standard of care (usual care) for primary and secondary prevention of cardiovascular disease at St Michael’s Hospital, Toronto, Ontario, Canada. As part of this iKT approach to enhance the adoption of the Portfolio Diet, it is important to ensure that the PortfolioDiet.app meets the needs of its end users. The population of end users for the application includes adult patients at risk for cardiovascular disease and clinical staff who may wish to learn more about the diet or want to recommend the PortfolioDiet.app to their patients. By undertaking user-centered evaluations, the needs of the target population can be identified, leading to improved uptake of the application.

### Quality Improvement Initiatives

Quality improvement (QI) initiatives offer an opportunity to optimize and test current clinical tools and are a proven method to improve patient care [18]. These initiatives are especially important when the clinical tool is an app because usability problems have been identified as a major obstacle in the adoption of health apps and have been associated with attrition [19,20]. By performing usability testing of health apps, problems related to ease of use can be identified before undertaking costly trials. Although regarded by many as an essential step in app development, usability testing of nutrition apps is less common in the literature, possibly leading to low user engagement and loss of effectiveness over time [21]. In a recent systematic review by König et al [22], usability was the most frequently identified barrier by participants for nutrition apps, underpinning the importance of usability testing in the development of nutrition therapy apps. This paper provides a description of our user testing approach to help inform research groups seeking to improve similar apps. Therefore, the objective of this project is to undertake and describe user testing to inform modifications to the PortfolioDiet.app as part of ongoing engagement in QI.

## Methods

### System Intervention

The PortfolioDiet.app is based on a nutrition therapy to manage dyslipidemia, the Portfolio Diet, that was demonstrated to be effective in individuals with hyperlipidemia [1,2]. The PortfolioDiet.app is a freely available web-based application that can be accessed on any smartphone or PC [23]. A web-based platform was chosen as the initial form to ensure that the application was accessible to patients. Although most Canadians have home internet or smartphone access (94% and 86%, respectively, in 2017) [24], having a web-based platform allows those patients who do not have home internet or a smartphone to access the application through public computers such those as in libraries. Ensuring accessibility was especially important, given the inner city community that St Michael’s Hospital serves. The PortfolioDiet.app is automated and patient facing. The application contains a variety of personalized elements to enhance and sustain patient education and engagement based on a 25-point Portfolio Diet score. These include elements preferred by health app users: an interaction-enabled dashboard, learning resources, gamification components, nudging, and so on [25]. The dashboard presents various summary statistics on adherence, such as total score, individual diet component score, and a 30-day score trend (Multimedia Appendix 1 shows screenshots depicting the various features on the dashboard of the application). The learning resources in the application include the Portfolio Diet infographic, recipes, tip sheets, and educational videos. The infographic provides a visual of the Portfolio Diet and its health benefits (Multimedia Appendix 2). The recipes were developed by registered dietitians according to the Portfolio Diet’s targets. The gamification components include star rewards, weekly quiz questions about the Portfolio Diet, and the Portfolio Diet score leaderboard. Users gain star rewards for each log-in of the day and for completion of weekly quiz questions.

### Design

**Figure 1:**
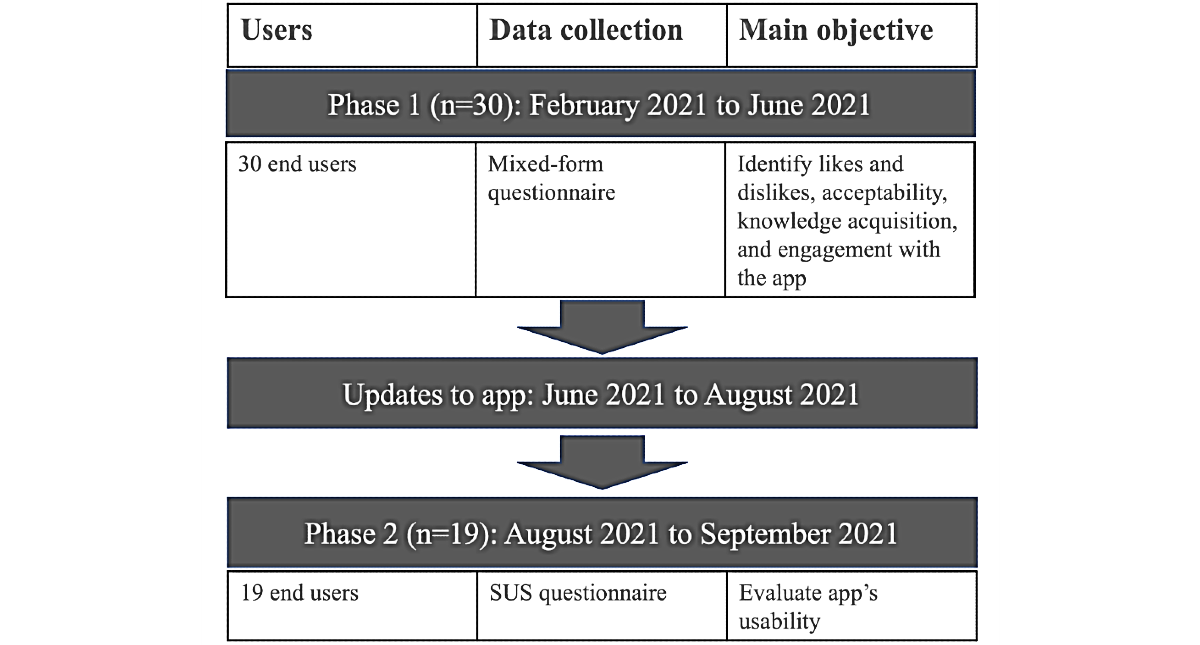
Project overview. SUS: System Usability Scale.

We performed a 2-phase QI project from February 2021 to September 2021 (Figure 1). Adult users from a number of areas were invited by email to participate in the testing of the application. Selective convenience sampling was used to generate a varied sample of previously identified end-user groups: patients with hyperlipidemia, family physicians and registered dietitians, the general public, and nutrition and medical students. For both phases, users were provided with the PortfolioDiet.app link and an instructional guide (Multimedia Appendix 3 shows example pages from the instructional guide) and asked to use the PortfolioDiet.app daily for 7 days. As the application is intended to be used over a long-term duration, a 7-day time frame was chosen to ensure that the users had sufficient time to experience each PortfolioDiet.app feature, such as the personalized weekly email reminders and accumulation of their daily scores displayed on the 30-day graph.

For phase 1, users were sent an email at the end of the 7 days, asking them to complete a mixed-form questionnaire and return it by email (Multimedia Appendix 4). The questionnaire was developed with experts in knowledge uptake evaluation. The purpose of the questionnaire was to evaluate the user’s perceived acceptability, knowledge acquisition, and engagement with the PortfolioDiet.app. The questionnaire collected both quantitative and qualitative data, including 2 open-ended questions. A mixed-form questionnaire allows for a more comprehensive collection of data on views and feedback from end users [26]. Open-ended questions were included to provide users an opportunity to identify strengths and limitations. In phase 2, the usability of the PortfolioDiet.app was measured using the System Usability Scale (SUS; Multimedia Appendix 5). The SUS is a validated usability questionnaire that has been used in clinical settings to assess the usability of various systems and tools [27,28]. The SUS includes 10 statements rated on a 5-point Likert scale. The Likert scale is a psychometric scale often used in psychology questionnaires and frequently applied in health, nutrition, and foods research as well as QI to assess the acceptability of systems and tools. These scales are often used to assess personality, attitudes, and behaviors.

The application users were asked to indicate their age range (<40 years, 40-60 years, >60 years) because age has been previously identified as an important covariate when assessing usability and is inversely correlated with the SUS score, whereas other characteristics such as gender have not [29]. Other than age range, no other demographic information from users was collected. During the QI project, the core team held weekly meetings to coordinate the application development process. User feedback from phase 1 was discussed within the research team during these weekly meetings. Modifications and updates to address user feedback were implemented in the PortfolioDiet.app and its supporting material before the initiation of phase 2. Although sample sizes of n=5 have previously been deemed acceptable for usability testing [30], a sample of at least 25 was decided upon to ensure a high level of problem detection [31]; therefore, with an anticipated response rate of 80% [32], a total of 30 users were invited. All 30 invitees accepted.

### Data Analysis

All open-ended responses from the questionnaire were collected and sorted manually into either limitations or strengths. Common comments (reported by ≥2 users) were identified and summarized. Representative quotations of common comments were included to improve the credibility of the findings, an approach recommended by Graneheim and Lundman [33]. All quantitative data were grouped and summarized as totals. A summary usability score was calculated (range 0-100) using the standard score conversion procedure for the SUS [27], with a score higher than 70 being considered acceptable [29]. Data were expressed as mean (SD).

### Ethics Approval

This project was formally reviewed by institutional authorities at Unity Health Toronto and deemed to require neither research ethics board approval nor written informed consent from participants.

## Results

### Phase 1

For phase 1, a total of 30 users provided feedback from February 2021 to June 2021, with 20 (66%) users aged <40 years, 6 (20%) aged 40-60 years, and 4 (13%) aged >60 years. The response rate for the questionnaire was 100% (30/30). Table S1 in Multimedia Appendix 6 presents the results of the quantitative responses of phase 1. Of the 30 users, 29 (97%) said the PortfolioDiet.app increased their knowledge of the Portfolio Diet, demonstrating that perceived knowledge acquisition was high. Most of the users reported that the application influenced or changed their food choices (24/30, 80%) and that they would use the application daily (20/30, 67%) or weekly (9/30, 30%), demonstrating a high level of engagement with the application. In addition, users ranked the infographic and the tip sheets as the first and second highest features that helped them learn about the Portfolio Diet. Users ranked the star rewards (a gamification component) and recipes as the first and second highest features that supported their interest and engagement in using the application. Most of the users responded that the application was easy to use (26/30, 87%) and it was easy to navigate between the applications functions (28/30, 93%), demonstrating acceptability. Common comments from ≥2 users are summarized as representative quotations in Textbox 1 (a full report of all comments can be found in Table S2 in Multimedia Appendix 6).

Feedback from phase 1 revealed several opportunities for improvement of the PortfolioDiet.app and its content. The user feedback was reviewed by the team during weekly meetings, and modifications to the second prototype of the application were made to address the common comments. Subsequently, usability of the updated application prototype was assessed in phase 2.

**Qualitative data from users in phase 1.**

**Representative quotations of feedback on the PortfolioDiet.app after using it for 7 days.**

1. Limitations and suggestions for improvement
  “I got more familiar with the food items in each category. I might have learned more but I didn’t realize at first that there was anything important in the ‘Learn’ section.”
  “I found some portions to be very large.”
  “...one thing I did not enjoy was the lack of information about plant sterols and where to find/purchase these.”
  “The app did not accurately record my average scores.”
  “It would be nice not to have to log in each and every time, if the app could remember my login info.”
  “It would be helpful to possibly add a video or a step-by-step guide that pops up when you first enter the app. Otherwise, it felt like I had to search for the diet outline and recipes myself.”
  “Would prefer an actual app, and not doing it via web browser.”
2. Positives and strengths
  “I learned a lot about which foods are part of the diet as well as quantities needed for one serving”
  “The resources gave good summaries of the Portfolio Diet. I was unaware of the Diet prior to beginning using the app so it was a good introduction. The tip sheets and recipes were very helpful.”
  “I found the front page most useful by allowing me to see where I am not meeting the daily targets, and where and what I still need to eat for the day.”
  “I loved how easy it was to enter information into the app and the progress bar really helped me visualize my progress.”
  “The recipe booklet offered many great and creative meal ideas, and I can personally say I have used it since, and will continue to use it moving forward.”

### App Updates Based on Themes

#### Navigation to Resources: Theme 1

Users reported navigation challenges with the phase 1 application prototype. For example, some users were unsure how to initially navigate through the PortfolioDiet.app to find resources. Although a PDF instructional guide was provided to all users, this may have not been the most suitable format for communicating with all user types. Therefore, several short videos were created to help supplement the PDF instructional guide, resulting in 9 tutorial videos lasting from 1 minute to 3 minutes to familiarize users with the PortfolioDiet.app and its functions.

#### Navigation to Settings: Theme 2

Multiple users noted concerns with the food portions within the application; a user commented: “I found some portions to be very large.” The application automatically starts all users on the 2000 kcal per day diet. Although instructions for users on changing calorie targets were provided through the PDF instructional guide, it may have not been intuitive to users that the fruit icon at the top of the home page would lead them to their account settings. Therefore, to help users navigate to their account settings, the fruit icon was changed to an *Account* button (Multimedia Appendix 7 shows the screenshots of the application changes). In addition, a short instructional video was created that explained to users how to correctly select their appropriate kcal target per day in the account settings of the application.

#### Plant Sterol Familiarity: Theme 3

Users expressed limited knowledge of plant sterols. As plant sterols are 1 of 5 major components of the Portfolio Diet, it is important that patients feel informed and comfortable incorporating plant sterols into their diet. It was decided that the creation of an evidence-based educational resource was critical to helping communicate the health benefits of plant sterols to patients and clinical staff. Therefore, a plant sterol tip sheet was developed and added to the updated version of the application (Multimedia Appendix 7).

#### Saving Issue Leading to Point Inaccuracy: Theme 4

Of the 30 users, 2 (7%) expressed concerns regarding inaccuracies in their 25-point Portfolio Diet score calculated by the application. An investigation with the application development team determined that after 45 minutes the application was not connecting with the server and not saving food entries for some users. To address this, a logout notification was added to inform users when to refresh and log back into the application (Multimedia Appendix 7).

#### Logout Frustration: Theme 5

Users expressed frustration with the application automatically logging them out after 45 minutes of inactivity. To help reduce user frustration, the automatic logout was extended to 21 hours as a balance between user experience and personal health data security.

#### Opportunities for Future Improvements: Themes 6 and 7

Although many improvements to the PortfolioDiet.app were made based on phase 1 feedback, certain user feedback remained challenging to address in the short term (Textbox 1: themes 6 and 7). Users suggested the addition of pop-up windows to help with initial navigation to important areas. Although the QI team agreed with the benefits of pop-ups, this proved challenging to implement and was considered lower priority than other key application issues identified by users. In addition, users expressed interest in, or preference for, a mobile app over the current web-based platform. A web-based platform was chosen as the initial form to ensure accessibility of the application. Future work to enhance the adoption of this tool will include the development of an iOS app and an Android app for mobile use as well as the integration of pop-ups to further engage participants with features and resources.

#### Recipes: Theme 12

The enjoyment of the recipes was the most commonly praised feature by users and was the second favorite application feature supporting engagement with the PortfolioDiet.app (Table S1 in Multimedia Appendix 6). Therefore, the recipe bank was expanded from 53 to 70 recipes and culinary students were engaged in this work to expand the cultural diversity of the recipes. To enhance usability, the downloadable PDF recipe book was converted into a filterable recipe webpage, allowing users to filter recipes by each of the Portfolio Diet categories. Recipes were also made filterable by type of meal (eg, breakfast, lunch, dinner, and snack), preparation difficulty level (eg, beginner and intermediate), and preparation time (eg, quick; Multimedia Appendix 7).

### Phase 2

In phase 2, a total of 19 users completed the SUS from August 2021 to September 2021, with 11 (58%) users aged <40 years, 5 (26%) aged 40-60 years, and 3 (16%) aged >60 years. The response rate was 79% (19/24). Nearly half of the participants (9/19, 47%) were new to using the application. The rest were previously users in the phase 1 testing who were reapproached and asked to again use the updated application for 7 days. The participating users gave the application a mean SUS score of 85.39 (SD 11.47). Full responses to the individual SUS items are shown in Table S3 in Multimedia Appendix 6. Examination of the responses to the individual SUS items showed that most users thought that they would not need the support of a technical person to use the application (average rating of 1.11, SD 0.32, out of 5, where 1=strongly disagree and 5=strongly agree), they thought that the application was easy to use (4.47, SD 0.84), they believed that most people would learn to use the application very quickly (4.68, SD 0.67), and they felt confident using the application (4.32, SD 0.76). There were 2 questions where, although most of the users agreed that they would use the application frequently (3.58, SD 0.90) and that the various functions in the application were well integrated (3.79, SD 1.32), these scores averaged closer to a neutral rating; therefore, updates to further improve application engagement and application function integration will be a focus during the next application revision.

## Discussion

### Principal Findings

The result of this QI project is a clinical tool that better meets the needs of end users. Through this 2-phase QI project, user feedback was collected and common issues and strengths were identified. The feedback was then used to make modifications to the application. Users considered the updated PortfolioDiet.app as acceptable, giving it a mean SUS score of 85.39 (SD 11.47), which is above the usability quality benchmark threshold score of 70.

In phase 1, the PortfolioDiet.app was found to increase users’ perceived knowledge of the Portfolio Diet and to influence their perceived food choices. Responses to open-ended questions revealed common issues and suggestions related to challenges with navigating to (1) resources and (2) profile settings, (3) limited information on plant sterols, (4) inaccuracies in points, (5) timed-logout frustration, (6) request for step-by-step pop-up windows, and (7) request for a mobile app version. When looking at positive feedback, the enjoyment of the recipes was the feature most commonly praised by users. Between the project phases, 6 key modifications were made to the PortfolioDiet.app to incorporate user feedback. In phase 2, the participating users gave the updated PortfolioDiet.app a mean SUS score of 85.39 (SD 11.47). The remaining suggestions to be addressed from phase 1 (Textbox 1: themes 6 and 7) should be prioritized in the next update of the application. Moreover, as identified in the SUS findings in phase 2, the focus should be on engagement and function integration to improve the application’s overall usability. To increase engagement, adding social features to the PortfolioDiet.app is recommended. Social features can enhance the benefits of gamification components in engaging users. Patel et al [34] found that a web-based intervention with social support and competition increased physical activity in individuals with type 2 diabetes compared with a control intervention consisting of feedback alone.

### Comparison With Previous Work

To our knowledge, this is the first QI initiative undertaken with a nutrition therapy application. There is a paucity of literature focused on QI initiatives with health apps in clinical practice. Although trials investigating the benefits of health apps are common, their findings are inconsistent and the details of their QI initiatives are unclear or not reported. The totality of evidence for web-based applications targeting risk factors for cardiovascular disease found beneficial effects on blood pressure, glycated hemoglobin level, LDL-C, body weight, and physical activity compared with standard of care alone in a systematic review and meta-analysis of 47 randomized controlled trials [15]. However, the evidence for the use of mobile apps to improve health outcomes, although positive, was considered weak based on a recent systematic review and meta-analysis [35]. When looking specifically at mobile apps targeting nutrition-related behaviors, Villinger et al [21] found benefits on both nutrition behaviors and nutrition-related health outcomes; however, these benefits were only found in short-term studies lasting for <6 months. The lack of benefits found in longer-term nutrition app studies may be related to low user engagement because of app usability barriers [22]. Nutrition apps are particularly susceptible to usability issues because they require the user to manually enter food data to provide the user with feedback compared with apps that link to accelerometers and other wearable health devices, such as physical activity apps. These inconsistent findings demonstrate the importance of QI and usability testing of health apps before conducting costly trials.

Previous studies have assessed the usability of digital dietary assessment tools, but these tools were only intended for dietary intake assessment and not for delivering nutrition therapies [36,37]. Usability testing of other lifestyle therapies has been conducted, including a web-based exercise program for older adults (mean SUS score of 84.2, SD 13.3) [38] and a comparison of 2 web-based interventions to increase physical activity, with mean SUS scores of 61.7 (SD 10.8) and 62.5 (SD 11.1) [39]. Another study assessed usability testing of a lifestyle intervention app in patients with type 1 or type 2 diabetes and found a mean SUS score of 62.0 (SD 18.0) [40].

### Strengths and Limitations

The purpose of this QI project was to conduct initial testing of the PortfolioDiet.app and to integrate a diverse group of end users in the development and testing of the application. By collecting both qualitative and quantitative data, this project allowed for a more comprehensive collection of data on the views of, and feedback from, end users. The data collected through this QI project identified several important issues with the previous application version that were able to be addressed and also provided direction for future development. The expansion of the tailored recipes may be especially important because of concerns in the literature regarding the nutritional content of internet recipes [41]. The COVID-19 pandemic has shown that maintaining care at a distance was not only essential, but must also be done well. The resulting application would strongly support distance care both in times of the pandemic and beyond.

A limitation of our study was that we used a convenience sample of users. Although convenience samples have been previously found to increase the risk of bias to favor the intervention, we attempted to limit the bias by purposefully reaching out to a broad range of users (patients with hyperlipidemia, family physicians and registered dietitians, the general public, and medical and nutrition students), resulting in an assorted sample. This assorted sample of users may have allowed for more barriers to be identified. The need for more information on plant sterols may have not emerged with a sample of informed patients and staff from a specialist lipid clinic.

Another limitation is that the SUS was not specifically designed to evaluate therapeutic health apps and is recommended to be combined with other usability metrics. Although there are various methods available to test the usability of therapeutic apps, the SUS is commonly used in the literature [42-44], which allows for comparisons with other therapeutic lifestyle intervention apps. In addition, the SUS uses an intuitive 100-point scale for the score, allowing findings to be easily communicated to those outside of the usability field. Another benefit of the SUS is that it can be completed by users in a short period of time with 10 questions. Other questionnaires developed to assess health apps are longer, increasing user response burden. Future assessments of the mobile version of the PortfolioDiet.app will include other questionnaires more specific to mobile health apps, such as the user version of the Mobile Application Rating Scale questionnaire [45].

### Conclusions

The continued consultation with knowledge users throughout the development and testing process of the PortfolioDiet.app aligns with participatory research or iKT approaches [17]. The result of this QI project is a clinical tool that better meets the needs of end users. Although the therapeutic benefits of the Portfolio Diet are well established and the PortfolioDiet.app was demonstrated to increase knowledge of the Portfolio Diet and is usable, the impact of the PortfolioDiet.app on LDL-C and cardiovascular risk reduction is unknown. Therefore, the next step will be to evaluate the utility of the PorfolioDiet.app in primary care settings in a clinical trial.

## Appendix

Multimedia Appendix 1 Screenshots depicting the various features on the dashboard of the application.

Multimedia Appendix 2 The Portfolio Diet infographic.

Multimedia Appendix 3 Example pages from the instructional guide (navigation and progress).

Multimedia Appendix 4 Mixed-form feedback questionnaire (phase 1).

Multimedia Appendix 5 System Usability Scale (phase 2).

Multimedia Appendix 6 Tables showing full quantitative responses (phase 1), full qualitative responses broken up by limitations and strengths (phase 1), and scores for individual System Usability Scale items (phase 2).

Multimedia Appendix 7 Screenshots depicting updates made to the application based on user feedback.

## Abbreviations

iKT: integrated knowledge translation
LDL-C: low-density lipoprotein cholesterol
QI: quality improvement
SUS: System Usability Scale
