# Supplementary material for: A Web-Based Health Application to Translate Nutrition Therapy for Cardiovascular Risk Reduction in Primary Care (PortfolioDiet.app): Quality Improvement and Usability Testing Study
Source: JMIR Hum Factors. 2022 Apr 21;9(2):e34704. doi: 10.2196/34704 (PMC9073604; doi:10.2196/34704)
Supplement: Multimedia Appendix 1 [file humanfactors_v9i2e34704_app1.pdf]

Multimedia Appendix 1: Application Screenshots Depicting the Various Features on the Dashboard of the Application

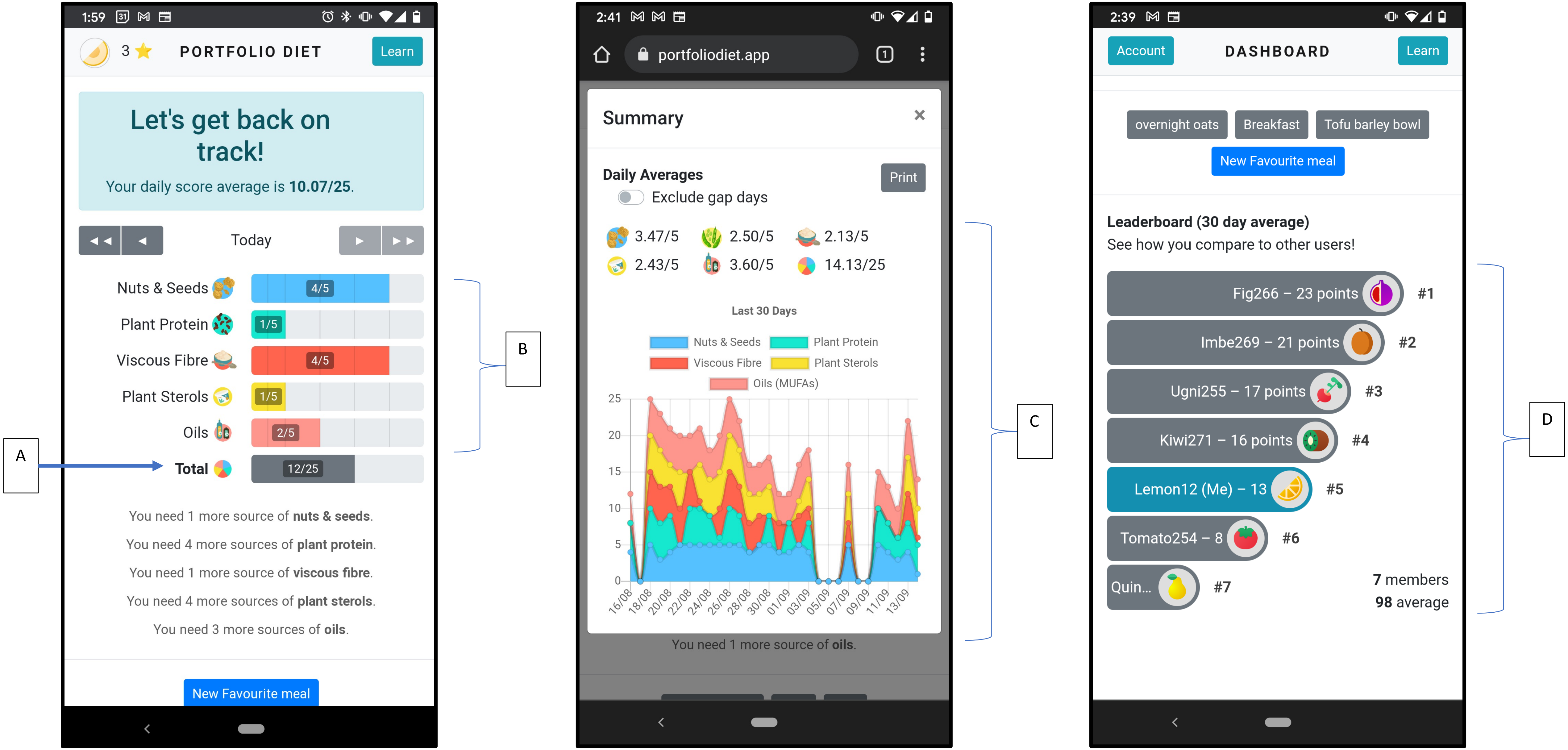

Dashboard summary statistics on adherence: A, total Portfolio Diet score; B, individual Portfolio Diet category scores; C, trend; D, leaderboard

This is a Multimedia Appendix to a full manuscript published in the J Med Internet Res.
