# Supplementary material for: A Web-Based Health Application to Translate Nutrition Therapy for Cardiovascular Risk Reduction in Primary Care (PortfolioDiet.app): Quality Improvement and Usability Testing Study
Source: JMIR Hum Factors. 2022 Apr 21;9(2):e34704. doi: 10.2196/34704 (PMC9073604; doi:10.2196/34704)
Supplement: Multimedia Appendix 2 [file humanfactors_v9i2e34704_app2.pdf]

— THE —

PORTFOLIO DIET

An evidence-based eating plan for lower cholesterol

WHAT IS THE PORTFOLIO DIET?

EGGPLANT · CHICKPEAS · VEGGIE BURGER · ALMONDS · SOY MILK

WALNUTS · TOFU · PLANT STEROL MARGARINE · OATMEAL · BEANS

The portfolio diet is a way of eating that evidence has shown can help lower cholesterol and your risk of heart disease. Instead of focusing on what you can't eat, the Portfolio diet is about what you can add to your menu!

The diet includes a **"portfolio"** of plant foods that you can choose from.

Research shows that medications and diet both work to lower your cholesterol. Medications can be more effective and easier, but some people don't want to take medications, cannot tolerate the side effects, or want to combine a nutritious diet with medications.

HOW DOES IT WORK?

The Portfolio diet is exactly as it sounds. It takes a few dietary patterns that have been shown to lower cholesterol and puts them together. To lower your cholesterol, you can "invest" in any one pattern, or some of them, or all of them.

NUTS, LEGUMES, CEREALS

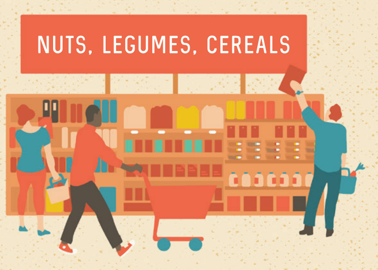

FRUITS & VEGETABLES

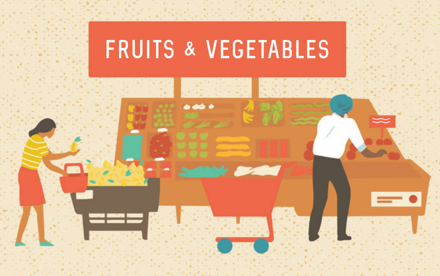

MEAT ALTERNATIVES

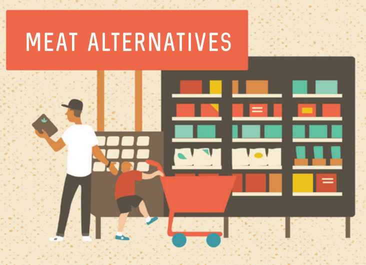

WHAT DOES THE PORTFOLIO DIET LOOK LIKE?

Expected LDL-Cholesterol lowering:

1 NUTS 45g DAILY

All nuts are good for your heart and contrary to concerns do not contribute to weight gain. Add nuts as a snack between meals, adding to salads, cereals, or yogurt. Trying nut butter on your toast is an option. 45g is about a handful of nuts. If allergic to peanuts or tree nuts, try seeds.

MIXED NUTS · ALMONDS · PEANUTS · NUT BUTTERS · PISTACHIOS · WALNUTS

5 - 10%

2 PLANT PROTEIN 50g DAILY

This is the most challenging component of the Portfolio diet. Start by trying to get 25g daily. Consider replacing milk with soy milk, try tofu, soy nuts and beans. .

CHICKPEAS · PEAS · TEMPEH · VEGGIE BURGER · TOFU · SOY BEANS · LENTILS · BEANS · VEGGIE DOG · SOY MILK · SOY DELI SLICES

5 - 10%

3 VISCOUS (STICKY) FIBRE 20g DAILY

Aim to eat 2 servings of oatmeal, beans, lentils, and chickpeas a day. Replace bread with rye or pumpernickel or oatcakes. Eat at least 5 servings of fruit and vegetables every day. Aim to eat 2 servings per day of oatmeal, barley, or cereals enriched with psyllium or oat bran. Replace white bread with whole grain oat breads. Put oat bran or psyllium into smoothies. Eat at least 5 servings per day of vegetables (eggplant, okra) and fruit (apples, oranges, berries) high in viscous fibre.

APPLE · OKRA · EGGPLANT · PSYLLIUM · STRAWBERRIES · OATMEAL · OAT BRAN CEREAL · BARLEY

5 - 10%

4 PLANT STEROLS 2g DAILY

These occur naturally (soyabean, corn, squash, etc.) but to get this amount of sterol you will require fortified foods such as spreads, juices, yogurt, milk and even supplements as part of a meal.

PLANT STEROL MARGARINE · OILS · JUICES · YOGURT

PLANT STEROL FORTIFIED

5 - 10%

TOTAL: ~30%

Statins, the most effective class of cholesterol-lowering medications, reduce cholesterol by 20-60%

IT'S NOT ABOUT ONE BIG CHANGE. IT'S NOT ALL OR NOTHING. JUST START BY INTRODUCING ONE COMPONENT TO YOUR DIET AND BUILD FROM THERE.

THIS PORTFOLIO DIET IS FOR "REAL PEOPLE IN THE REAL WORLD"

– DR. DAVID JENKINS, CREATOR OF THE PORTFOLIO DIET

David JA Jenkins MD, PhD, DSc, **Cyril WC Kendall** PhD, **Lilisha Burris** MHSc, RD, **John L Sievenpiper** MD, PhD, FRCPC, **Michael F. Evans** MD, CCFP, **Emily Nicholas Angl** BSc

Canadian Cardiovascular Society — Leadership. Knowledge. Community.

The Joannah & Brian Lawson Centre for Child Nutrition, UNIVERSITY OF TORONTO

St. Michael's — Inspired Care. Inspiring Science.
