# Supplementary material for: A Web-Based Health Application to Translate Nutrition Therapy for Cardiovascular Risk Reduction in Primary Care (PortfolioDiet.app): Quality Improvement and Usability Testing Study
Source: JMIR Hum Factors. 2022 Apr 21;9(2):e34704. doi: 10.2196/34704 (PMC9073604; doi:10.2196/34704)

Multimedia Appendix 3: Example Pages from Instructional Guide (Navigation and Progress)

HOW TO NAVIGATE THE HOMEPAGE

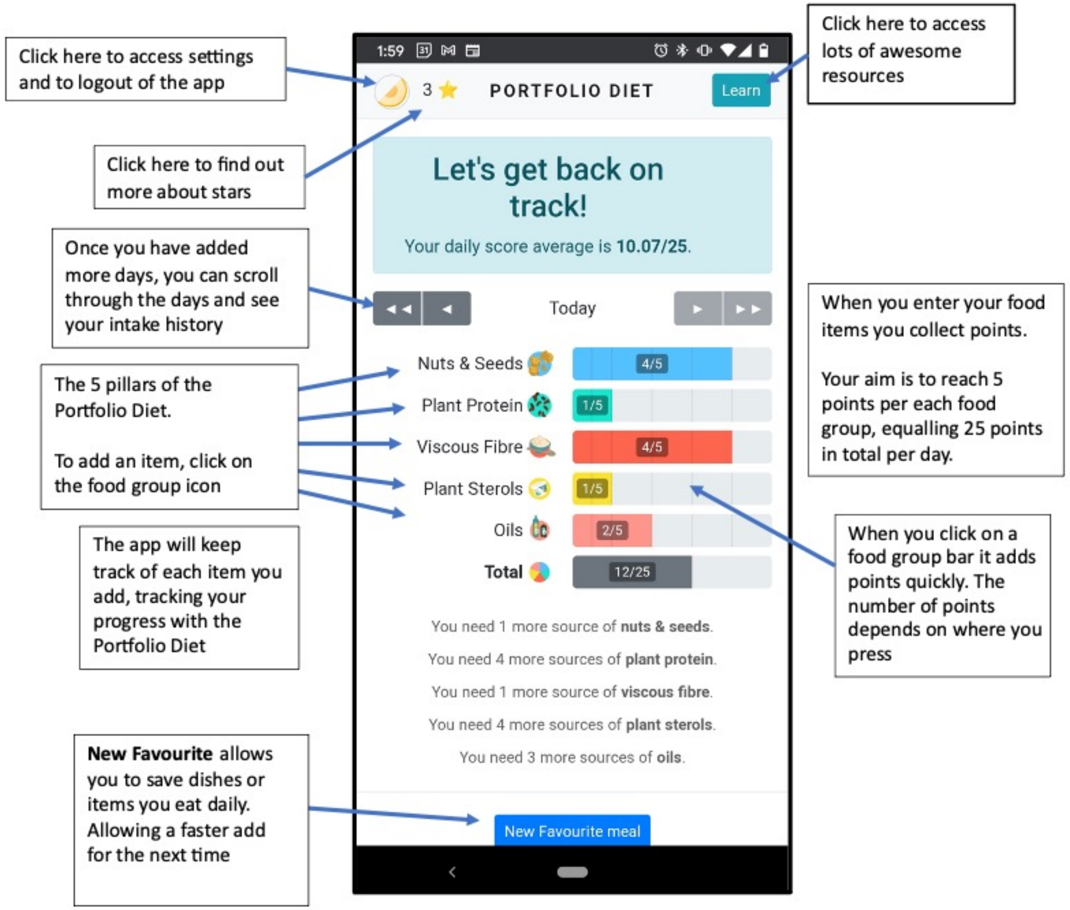

HOW TO VIEW YOUR RESULTS AND PROGRESS

- You can view your progress on the app homepage. Here, you will find your daily points intake. Your current daily intake is shown in a bar graph format.
- Scroll down the homepage and you can view the last 30 days of your daily point intake added to the app in a graph format
- Please refer to the *How to navigate the homepage* (page 7) section for details on where to locate these graphs

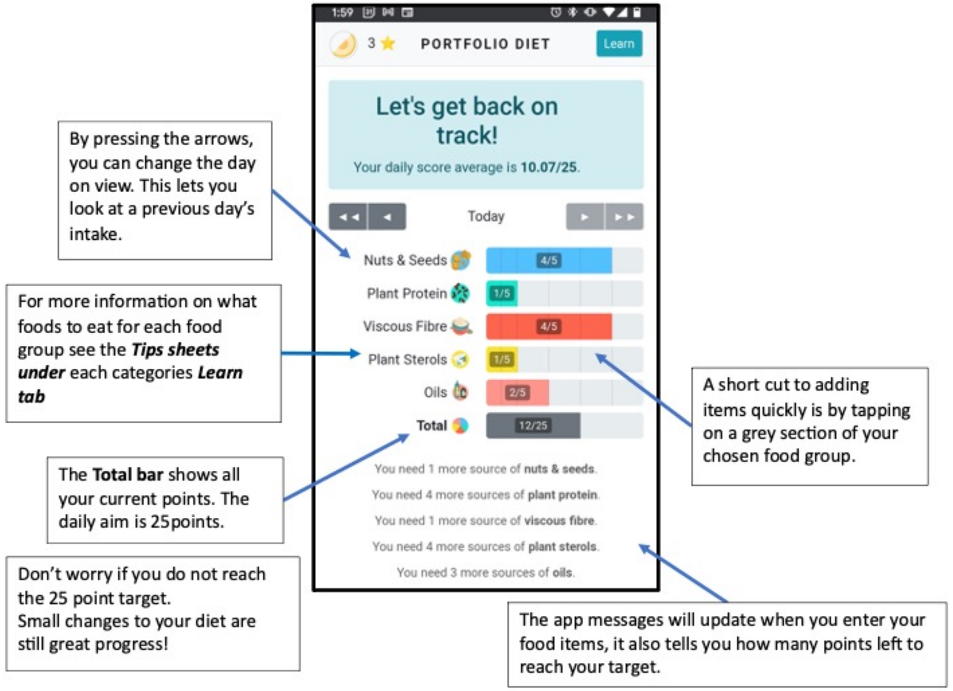

Supplement: Multimedia Appendix 3 [file humanfactors_v9i2e34704_app3.pdf]
