# Supplementary material for: A Web-Based Health Application to Translate Nutrition Therapy for Cardiovascular Risk Reduction in Primary Care (PortfolioDiet.app): Quality Improvement and Usability Testing Study
Source: JMIR Hum Factors. 2022 Apr 21;9(2):e34704. doi: 10.2196/34704 (PMC9073604; doi:10.2196/34704)
Supplement: Multimedia Appendix 4 [file humanfactors_v9i2e34704_app4.docx]

Multimedia Appendix 4: Mixed-form feedback questionnaire (phase 1) **Please answer the following questions below and then send it back to us by email. We hope to get your honest feedback about your experience with using the Portfolio Diet App. Your feedback will be used to improve the Portfolio Diet App for future research. 

If you are uncomfortable answering any of the questions, please feel welcome to skip them by writing *NA*.**
1. Did you increase your knowledge about the Portfolio Diet while using the Portfolio Diet app?  Please add an “X” in the brackets.

[    ] Yes

[    ] No

[    ] Not sure

2. Please expand on your answer above, and write your answer below each question.

           If yes, what did you learn?

            If no, why do you think the app did not increase your knowledge?

3. Did the app influence/change your food choices?

[    ] Yes

[    ] No

4. Was there anything you particularly enjoyed or did not like about the app? Please write your answer below.


5. Which app characteristic helped you learn about the diet the most? 
[    ] recipes

[    ] tip sheets

[    ] videos 
[    ] infographic

6. Which app characteristic supported your interest/ engagement in using the app the most? 

[    ] star rewards

[    ] recipes

[    ] tip sheets

[    ] videos
[    ] email reminders
[    ] 30-day points graph

[    ] leaderboard

7. Was the app easy to use?

[    ] Yes

[    ] No

8. Were you able to navigate between functions of the app easily?

[    ] Yes

[    ] No

9. How often would you use the app?

[    ] Every day

[    ] Weekly

[    ] Monthly

[    ] Rarely

[    ] Never

10. In your home, who purchases the food most often?  Please add an “X” in the brackets. Choose all that apply.

[    ] I do 
[    ] my parent(s)/ caretaker

[    ] my children

[    ] my roommate 
[    ] my spouse or partner

[    ] other, please specify: [          ]

11. In your home, who makes the meals most often?  Please add an “X” in the brackets. Choose all that apply.

[    ] I do 
[    ] my parent(s)/ caretaker

[    ] my children

[    ] my roommate 
[    ] my spouse or partner

[    ] other, please specify: [          ]

12. What best describes your age?

[    ] <40y

[    ] 40-60y

[    ] >60y

13. Please provide the email address associated with your app account:

14. Please feel free to leave any other comments:

--- Thank you! ---
--- End of questionnaire ---
