# Supplementary material for: A Web-Based Health Application to Translate Nutrition Therapy for Cardiovascular Risk Reduction in Primary Care (PortfolioDiet.app): Quality Improvement and Usability Testing Study
Source: JMIR Hum Factors. 2022 Apr 21;9(2):e34704. doi: 10.2196/34704 (PMC9073604; doi:10.2196/34704)
Supplement: Multimedia Appendix 5 [file humanfactors_v9i2e34704_app5.docx]

# Multimedia Appendix 5: System Usability Scale (phase 2)

**Please answer the following questions below and then send it back to us by email. We hope to get your honest feedback about your experience with using the Portfolio Diet App. Your feedback will be used to improve the Portfolio Diet App for future research.**

**The following 10 questions are asking about how easy you found using the app was. Please add an “X” in the brackets.** Please do not think about the question for a long time, just provide your first response to each question. If you feel you cannot respond to a particular question, please mark the centre box of the scale.

1. I think that I would like to use this app frequently (often)

Strongly disagree [    ] [    ] [    ] [    ] [    ] Strongly agree

2. I found the app unnecessarily complex

Strongly disagree [    ] [    ] [    ] [    ] [    ] Strongly agree

3. I thought the app was easy to use

Strongly disagree [    ] [    ] [    ] [    ] [    ] Strongly agree

4. I think that I would need the support of a technical person to be able to use this app

Strongly disagree [    ] [    ] [    ] [    ] [    ] Strongly agree

5. I found the various functions in this app were well integrated (linked together)

Strongly disagree [    ] [    ] [    ] [    ] [    ] Strongly agree

6. I thought there was too much inconsistency (mismatch) in this app

Strongly disagree [    ] [    ] [    ] [    ] [    ] Strongly agree

7. I would imagine that most people would learn to use this app very quickly

Strongly disagree [    ] [    ] [    ] [    ] [    ] Strongly agree

8. I found the app very cumbersome (hard) to use

Strongly disagree [    ] [    ] [    ] [    ] [    ] Strongly agree

9. I felt very confident using the app

Strongly disagree [    ] [    ] [    ] [    ] [    ] Strongly agree

10. I needed to learn a lot of things before I could get going with this app

Strongly disagree [    ] [    ] [    ] [    ] [    ] Strongly agree

11. What best describes your age?

[    ] <40y

[    ] 40-60y

[    ] >60y

12. Please provide the email address associated with your app account:

--- Thank you! ---
--- End of questionnaire ---
