# Supplementary material for: A Web-Based Health Application to Translate Nutrition Therapy for Cardiovascular Risk Reduction in Primary Care (PortfolioDiet.app): Quality Improvement and Usability Testing Study
Source: JMIR Hum Factors. 2022 Apr 21;9(2):e34704. doi: 10.2196/34704 (PMC9073604; doi:10.2196/34704)
Supplement: Multimedia Appendix 6 [file humanfactors_v9i2e34704_app6.docx]

# Multimedia Appendix 6: Tables showing full quantitative responses (phase 1), full qualitative responses broken up by limitations and strengths (phase 1), and scores for individual System Usability Scale items (phase 2).

Table of Contents

[Supplement Table S1: Full quantitative responses (phase 1) 1](#_Toc92275193)

[Supplement Tables S2: Full qualitative responses broken up by limitations and strengths (phase 1) 1](#_Toc92275194)

[Supplement Table S3: Scores for individual SUS items (phase 2) 12](#_Toc92275195)

# **Supplement Table S1:** Full quantitative responses (phase 1)

| **Question,** Phase 1 (n=30) | **Response** |
| --- | --- |
| Did you increase your knowledge about the Portfolio Diet while using the Portfolio Diet app? (%; yes:no) | 97:3 |
| Did the app influence/change your food choices? (%; yes:no) | 80:20 |
| Which app characteristic helped you learn about the diet the most? (N users) | Infographic=16, tip sheets=7, recipes=3, videos=2, missing=2 |
| Which app characteristic supported your interest/ engagement in using the app the most? (N users) | Star rewards=8, recipes=7, 30-day points graph=6, email reminders=3 leaderboard=2, videos=2, tip sheets=1, missing=1 |
| Was the app easy to use? (%; yes:no:missing) | 86:10:3 |
| Were you able to navigate between functions of the app easily? (%; yes:no:missing) | 93:3:3 |
| How often would you use the app? (N users) | Every day=20, weekly=9, rarely=1 |
| What best describes your age? (%; <40 years: 40-60 years: >60 years) | 67:20:13 |

# **Supplement Tables S2:** Full qualitative responses broken up by limitations and strengths (phase 1)

| **User** | **Diet Feedback** | | **Technical App Feedback** | |
| --- | --- | --- | --- | --- |
|  | Strengths | Limitations | Strengths | Limitations |
| 1 | I learned what the portfolio diet comprised of and the health benefits associated with the diet |  | I liked that the app encompassed a variety of information - from the information sheets to the videos and recipes, these resources helped me integrate the Portfolio foods into my own diet with relative ease. I also liked the concept of the leaderboard as this helped motivate me to maintain/improve my diet so it could fit into the Portfolio diet better. | Some improvements could be to make the portion sizes smaller (e.g. I only eat one small apple per day and ate ~100g eggplant, so the values I recorded in the app were an over estimation of my actual intake); and I wish there was a tip sheet and recipe sheet for plant sterols as I was the least familiar with this food category (if there was information to help me figure out why and how I should be using plant sterols over other fats like olive oil, or if plant sterols can replace X amount of butter for baking... that kind of information would be very helpful). I also would like to know more about the leaderboard - is it based off the points only, or do stars also count towards it (and are there benefits to the stars alone)? Finally I have one suggestion: if the portfolio foods are meant to be incorporated into the diet, it may be a good idea to provide a guide on how to cook key Portfolio foods (e.g. baking, boiling, pan-frying vegetables), how to incorporate foods together (e.g. mix-and-match visuals for inspiration to make salads/trail mixes/etc.), and how Portfolio foods can replace typical foods (e.g. X amount butter can be substituted for X amount plant sterols). Providing generalized cooking guides can help people to incorporate Portfolio foods into their diet with more flexibility based on their own tastes/preferences instead of relying on the recipes |
| 2 | The app did a good job of giving a brief summary of the components of the portfolio diet, its purpose, and possible recipe/meal suggestions that would fit within its guidelines. | In the tip sheet as well, there were some foods that fell into two categories (ex. chickpeas were in both plant protein and viscous fibre) so it would be helpful to clarify if something like that would be entered twice, or if you should just choose one. Along those lines, I think for many options like nuts and seeds or viscous fibre, many people are eating things like oat bars or nut/cereal bars for snacks and might have questions on where those would fit within these guidelines. |  | The app was fairly intuitive. I do think it would be helpful to possibly add a video or a step by step guide that pops up when you first enter the app. Otherwise, it felt like I had to search for the diet outline and recipes myself. |
| 3 | The app has great resources that taught me a lot about the portfolio diet including: the how to incorporate the key foods into my diet, the required serving sizes, plant based proteins, fun and delicious recipes (the muffins are great!), and how the food I put into my body impacts my overall health and well-being. |  | I love how colorful the app is! It makes it fun to log into each day. I also really liked how it broke down the categories I needed to incorporate more into my diet. | It would be nice to have the resources directly in the app rather than in a google doc. |
| 4 | I learned the basic nutritional groups in the Portfolio diet (nuts and seeds, plant protein, viscous fibre, plant sterols, oils) and some common foods in those groups. |  | I liked how the graph and leaderboard provide motivation to meet targets. | I didn't like the amount of screens/clicks it took to add foods eaten and thought the infographic could have been improved with more "bubbles" showing examples of foods within each nutritional group and less text/replacing text with bullet points. |
| 5 | To be frank, I was completely unaware of what the Portfolio Diet consisted of. Specifically, the “Learn” tab coupled with the instructional guide PDF were good onboarding feature for a novice such as myself. |  | The “Learn” tab was a great feature to add, and I found its contents very informative. Specifically, the recipe booklet offered many great and creative meal ideas, and I can personally say I have used it since, and will continue to use it moving forward |  |
| 6 | I learned how to get more plant-based proteins and viscous fibre into my diet. I learned about the different food categories that help to lower cholesterol. |  | I liked the tip sheets that were included; I found it helpful that you could add a new favourite meal to the app, so it’s easy to add up the points for things eaten frequently. The recipes gave me ideas for how to add fibre & plant proteins to my diet. | I had difficulty understanding the serving sizes in some cases (for example, I can’t fathom a scenario where I would eat 4 cups of eggplant!) |
| 7 | I learned about the specific items that count as plant sterols, I was unaware margarine was in this category. |  | I liked the drop down menus as well. | I didn’t like that it required me to log-in each time I used it, but this probably is just because it was a saved-from-internet app. I found the suggested ‘serving sizes’ to be confusing, although I assume this is a part of the portfolio diet. |
| 8 | I learned about the specifics of the diet plan, which I didn’t know previously. I learned about a number of recipes suitable for someone on the diet. |  | I liked the learning resources. I found those helpful for having a better understanding of the diet. And I really enjoyed the recipe collection. I also liked that gap days could be excluded from the scoring, as there was one day that I forgot to log my intake. |  |
| 9 | I learned a lot about which foods are part of the diet as well as quantities needed for one serving. | I was sometimes surprised by the quantities, for example 2 large apples as one serving. | I liked how much the app got me thinking about my food choices. I had the app in mind most every time I decided on meals, whether or not everything in my choices was in the direction of the diet. | Regarding what I didn’t like: It would be nice not to have to log in each and every time, if the app could remember my login info.  The app did not accurately record my average scores. When I first logged in to review the app, it counted that as day one even though I didn’t start until the next day. This aside, the daily averages were also otherwise quite inaccurate. One morning, I woke up with 3 points in the nuts and seeds section before I even started my day. |
| 10 | I know more about the food sources of viscous fibre and the recommended amount per day for each category. |  | I like how it is easy to put the consumption amount of each food category in the app. | However, it will be better if the information in “Learn” tab or “Recipes” tab can be showed in a pop-up instead of opening new tab. Also, it took e a little bit time to figure it out how the app works. I think many of the app’s users are seniors, so it will be great if we have a quick instruction for using the app when a user have just downloaded the app in the future. |
| 11 | Learned deeply the main components of the diet and how to incorporate them in some recipes. |  | The “level” reward system is interesting to create engagement. |  |
| 12 | Learned about different foods that qualify for the Portfolio diet, and some recipes to incorporate into my usual diet. |  | I enjoyed the graph that filled up as you met the components needed for each day. | I did not enjoy accidentally exiting the app by refreshing or using my phone’s back button out of habit. |
| 13 |  | I found some portions to be very large. For example, I had 1 cup of eggplants one night for dinner but I couldn’t input 0.25 of the portion listed. It might be helpful to have 0.25 as a portion option? | I loved how easy it was to enter information into the app and the progress bar really helped me visualize my progress. The layout is great and information is easy to find – my only suggestion would be to increase the size of the “Learn” button so that it’s more visible. | I also think the “Learn” button is a little small – I don’t really feel prompted to check out the resources because it’s in the top corner. I didn’t like how quickly the app timed me out. I found myself having to log in for every meal (at least on desktop). I was confused about the daily average number – mine was always in the decimals (e.g. 0.13/25). Maybe the calculations are a little off? |
| 14 | I was aware of what the Portfolio diet prior to using the app. However, I learned more about the foods that apply to the Portfolio Diet. I also learned, that although the Portfolio Diet isn’t restrictive, it is hard to consume all 5/5 food sources from each category. |  | I liked how aware I became with incorporating certain foods in my diet from the Portfolio Diet. I also liked the large variety of foods to choose from. | It would be nice if there was an option to input what type of “other foods” were consumed. |
| 15 | I am now consuming more regular nuts and seeds into my diet every morning. Understanding more about the viscosity of my food choices and how it affects my health. |  |  | A little complicated at first with so many instructions however once I started understanding the food sources I needed to consume more of, it became easier to make healthier choices during the week. |
| 16 | I learned how easy it was to incorporate foods from the diet into my daily habits. Many of the foods that are part of this diet I actually eat very often. While I am knowledgeable on these types of foods and already incorporate them into my diet, this app would be great for someone who is looking to learn more about the diet or needs that visual motivation to eat more of the types of foods. However, I LOVED the infographic! Very user friendly and easy to understand. A good reminder! |  |  | The only thing that I didn’t like was that the tips and recipes could have been easily missed being at the top. And taking you into a Google drive to then choose the sheet, this could be difficult for someone who isn’t as knowledgeable with Google drive. If it would be possible to bring you straight to the recipe sheet or tips sheet I think it would be more user friendly! |
| 17 | At first, I was not aware of the portfolio diet. After using the app and reading the infographic helped me to learn about the portfolio diet and what it is. On top of that I found the recipes to also be very helpful as it gives options on what a person can eat while being on the portfolio diet. I particularly found the infographic to be very helpful. It had a lot of information of the portfolio diet along with small illustrations which I really liked. |  |  |  |
| 18 | I was not at all aware of what the diet consisted. All I knew was that it was a plant-based diet. I now know which foods are considered part of the diet. I also learned about the health benefits of the diet. Fortunately, I am not too concerned about any of the health outcomes that the diet aims to address. | I also already have Soylent for one meal a day, so that part is easy. And I pretty much eat the same thing for breakfast and for snacks every day. So, the only thing I had to change from day to day was my entry for dinner. But, for someone who is eating different things all the time, or for someone who is eating at restaurants or taking out food, it would be much more challenging. |  | The hardest part of using the app is just figuring out if, what I ate that day, qualified under any of the categories. Other than that, the app took a bit of getting used to, but was generally easy to use. The main point of confusion is that it would log me out but not tell me I was logged out. Instead, certain buttons simply wouldn’t respond. For example, I could move between days, click on a category (such as “Nuts & Seeds”), click <Add>, choose “Other nuts” and specify the quantity. But, when I then hit the <Add> button, nothing happens. No message or anything. It took me while to figure out that I needed to kill the app (which I’m sure a lot of users may not know how to do), log in again and then redo my entry. |
| 19 | Having limited knowledge this app helped me with the types of foods in each group ant the amounts that I needed to eat . I found it interesting. I has equipped me with knowledge and tools to see out the remainder of days before next blood test, and beyond. | (far too much for me I struggled, the wind is another story) | I found the front page most useful by allowing me to see where I am not meeting the daily targets, and where and what I still need to eat for the day. I couldn’t have done better myself. |  |
| 20 | I got more familiar with the food items in each category. I want to explore and try the Recipes but haven’t yet had the chance. | I might have learned more but I didn’t realize at first that there was anything important in the “Learn” section. | I enjoyed watching my points accumulate. | Things I did not like: I was unable to install the homepage icon on my iphone as I use Firefox and not Safari. I did not enjoy the scrolling food items as I like seeing the whole list at once. The items are difficult to read as the entire line does not show up, often cutting off the serving size. In the desktop version I didn’t like the back and forward buttons for selecting days. If possible I’d recommend 7 buttons across the top labelled with the last 7 days. The most annoying thing is the app timing out after 45 minutes or so, requiring me to sign in again. This happened both on my iphone and on my laptop (where I also use Firefox). And, even worse, after signing back in some of my recent entries had not been saved, forcing me to try to remember and re-enter items. Ultimately I got fed up with this and stopped using the app. |
| 21 | I learned the various portion sizes required within the categories. It also reinforced what the categories are. | Unfortunately, with this knowledge it reinforced how difficult it is for me to get the required categories and portion sizes in. It had a negative response for me, in that it confirmed that I will never be able to get in the required amounts. It also prompted me to look up information on Plant Sterol, which seems quite controversial. I also learned that the Becel Plant Sterol Margarine is no longer available in Canada. I am not comfortable taking the supplements. |  | Although the app identifies portion size for example 7 nuts I found it also a bit annoying, too many clicks and almost having to do math…lol which is a weird step. For me it would be better to select nuts – and then enter the amount 14 nuts or 18 grams or ¼ cup and let the calculation of portion size be done in the back ground that when you press enter you see how close you are to the goal. Also when I go into the category, for example “Nuts and Seeds”, I then have to press “Add” and then I have to press “Select” and then I have to press the type of nuts and I cannot select mixed nuts, nor can I select more than one nut at a time. Then I have to click the amount in a separate box…which took me a few seconds the first time to understand. I kindly say, I found this super annoying, this is for clicks in before I have entered 14 nuts. I would have thought you pressed the category and the user would have been brought immediately to the choices – nuts…..also the precision of the types of nuts, walnuts or almonds or peanuts? The portion is all 9 grams so, very curious as to the distinction is it for research at your end that someone had better cholesterol control with almonds vs walnuts vs peanuts? I found that odd. I also found it confusing under plant protein, selection includes chickpeas ½ cup, what if I had 2 tablespoons of hummus, does that count for chickpeas? If so, I then need to calculated that 2 tablespoons is about 15mls, ½ cup is 125 mls so I can only select .5 of the portion which would be 62.5 mls so that really does not count or contributes to a protein so I suspect hummus does not count?? If it does I cannot add it would only allow me to put in 62.5 mls or ¼ cup. This would be the same problem if I added 2 tablespoons or chickpeas to a salad, I would not be able to put in the correct value. |
| 22 | Different ways to incorporate the portfolio diet, and learning different delicious recipes. |  | The videos, I found them well-made and intriguing and having the ability to see your progress along the days of usage. |  |
| 23 | Very helpful to have the list of foods I should actively incorporate into my daily meals. |  |  | Would prefer an actual app, and not doing it via web browser. |
| 24 | How much intake some nutrients in my diet. For example how many nuts I have intake. |  |  | I am getting used it +F26:F28how to add nutrients in my diet. Also, I am learning about my daily average. However, sometimes I have been confuse about my score. I cannot understand clearly |
| 25 |  |  | Prior to using the app, I was not aware of how much plant proteins/plant sterols play a significant role in helping to reduce cholesterol. I really liked the overall design/layout of the app and enjoyed the leaderboard- I found it very engaging and thought it was a very unique feature. |  |
| 26 |  |  | I learnt about the various ways your diet can help to lower low-density lipoprotein cholesterol. Previously, I also had not been aware of the wide variety of vegetable proteins that are available in grocery stores. The Portfolio Diet app videos on YouTube were helpful and I gained more understanding about what the diet consists of. I enjoyed the recipes and link to the cookbook; I find this will be very helpful for people who are unfamiliar cooking with plant-based proteins. I enjoyed the daily average graph that shows how well I’ve reached my goal for each week | However, one thing I did not enjoy was the lack of information about plant sterols and where to find/purchase these. |
| 27 | I learned more about the 5 categories of food that make up the Portfolio Diet as well as examples of food that fall under each category. Although, this is a dietary pattern I had previously learned about, actually completing it based on my diet enabled me to learn and remember more. |  | I like that it is very easy and straightforward to input my food items after each day, taking only a few minutes to do so. |  |
| 28 | The resources gave good summaries of the Portfolio Diet. I was unaware of the Diet prior to beginning using the app so it was a good introduction. The tip sheets and recipes were very helpful. I have not yet had time to read the book or view the videos. |  | It is helpful to know how one is meeting the requirements of the different categories. |  |
| 29 |  |  | I found out about the diet and wanted an app to track. All is new to me. | I wish it was an app for my phone. |
| 30 | I learned more about specific foods and where they fell into the pillars. Portion sizes that translate into “points” was a major learning point. I increased my knowledge about the diversity and variety of Portfolio foods. I increased my knowledge about the diversity and variety of Portfolio foods. |  | I really liked the supportive tone in the app and the celebration of eating the foods that align with the portfolio diet. I liked that is never felt shamed for not eating a portfolio food, rather I felt informed and educated as to what is a portfolio food |  |

#

# **Supplement Table S3:** Scores for individual SUS items (phase 2)

| **n = 19** | **Mean (SD)** |
| --- | --- |
| 1. I think that I would like to use this app frequently (often) | 3.58 (0.90) |
| 2. I found the app unnecessarily complex | 1.21 (0.42) |
| 3. I thought the app was easy to use | 4.47 (0.84) |
| 4. I think that I would need the support of a technical person to be able to use this app | 1.11 (0.32) |
| 5. I found the various functions in this app were well integrated (linked together) | 3.79 (1.32) |
| 6. I thought there was too much inconsistency (mismatch) in this app | 1.47 (0.84) |
| 7. I would imagine that most people would learn to use this app very quickly | 4.68 (0.67) |
| 8. I found the app very cumbersome (hard) to use | 1.37 (0.76) |
| 9. I felt very confident using the app | 4.32 (0.76) |
| 10. I needed to learn a lot of things before I could get going with this app | 1.53 (0.70) |
| **Overall SUS score** | **85.39 (11.46)** |

SUS Scores 1 = Strongly disagree; 5 = Strongly Agree
