# Supplementary material for: A Web-Based Health Application to Translate Nutrition Therapy for Cardiovascular Risk Reduction in Primary Care (PortfolioDiet.app): Quality Improvement and Usability Testing Study
Source: JMIR Hum Factors. 2022 Apr 21;9(2):e34704. doi: 10.2196/34704 (PMC9073604; doi:10.2196/34704)
Supplement: Multimedia Appendix 7 [file humanfactors_v9i2e34704_app7.pdf]

Multimedia Appendix 7: Images Depicting Updates Made to the Application Based on User Feedback

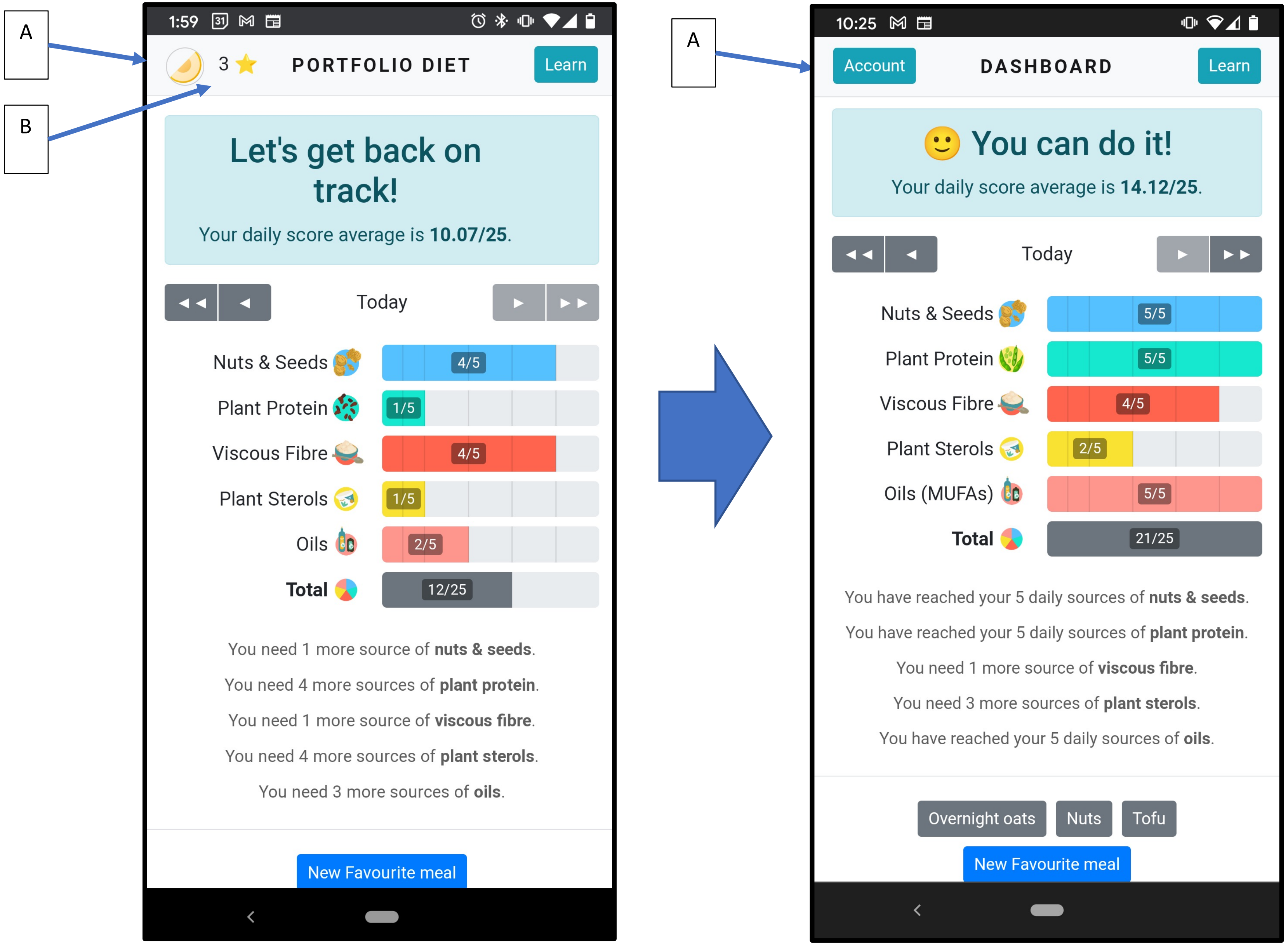

Navigation changes to main page: A, update to <Account> button to help navigate users to settings; B, removal of star rewards to account page to keep top bar clear and avoid clutter.

This is a Multimedia Appendix to a full manuscript published in the J Med Internet Res.

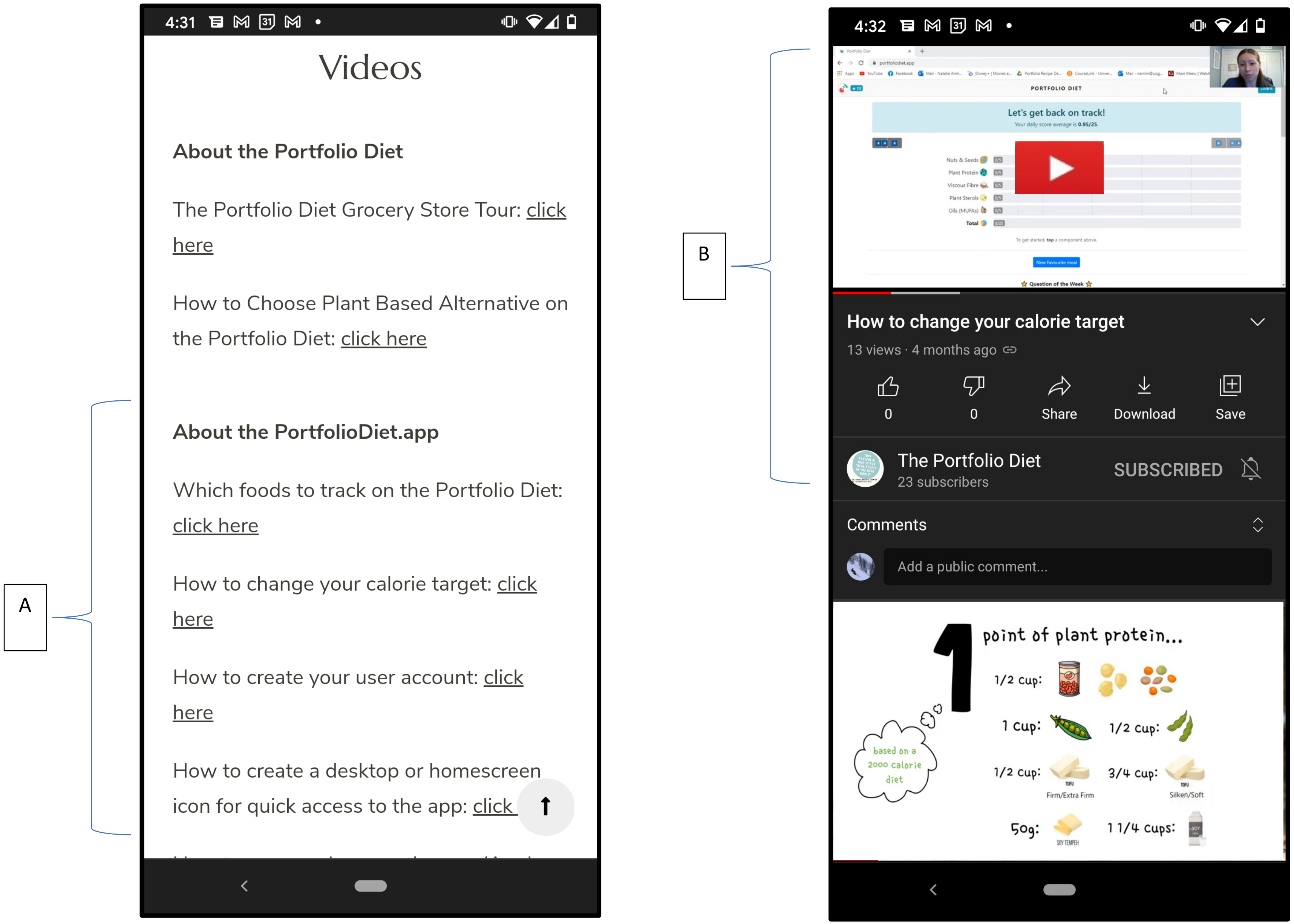

Creation of instructional videos to supplement the pdf instructional guide: A, instructional videos developed for key issues to supplement the pdf; B, example of video to help users change their calorie targe

This is a Multimedia Appendix to a full manuscript published in the J Med Internet Res.

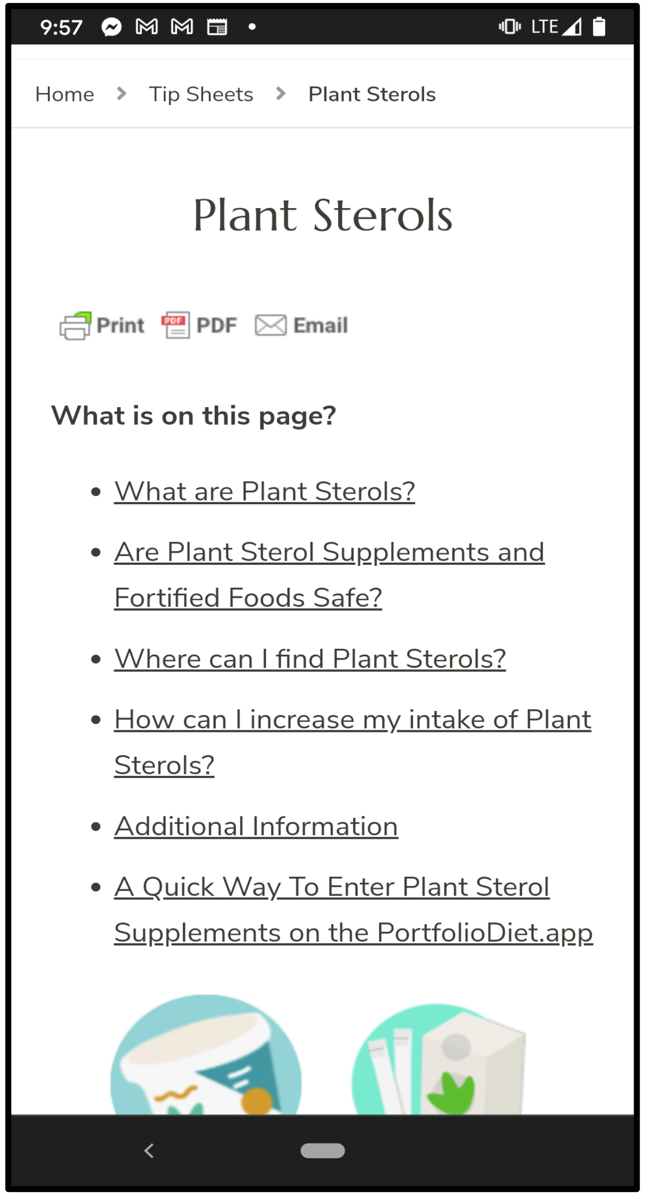

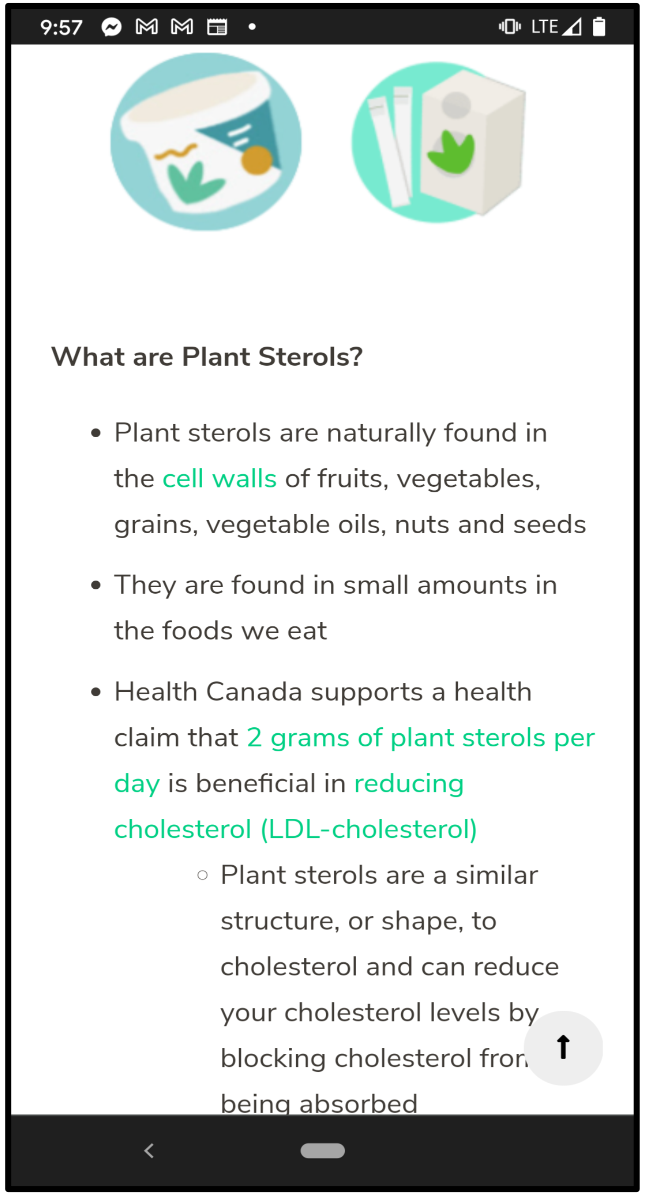

Creation of Plant Sterols tip sheet: A tip sheet was created to allow users access to evidence-based information on Plant Sterols

This is a Multimedia Appendix to a full manuscript published in the J Med Internet Res.

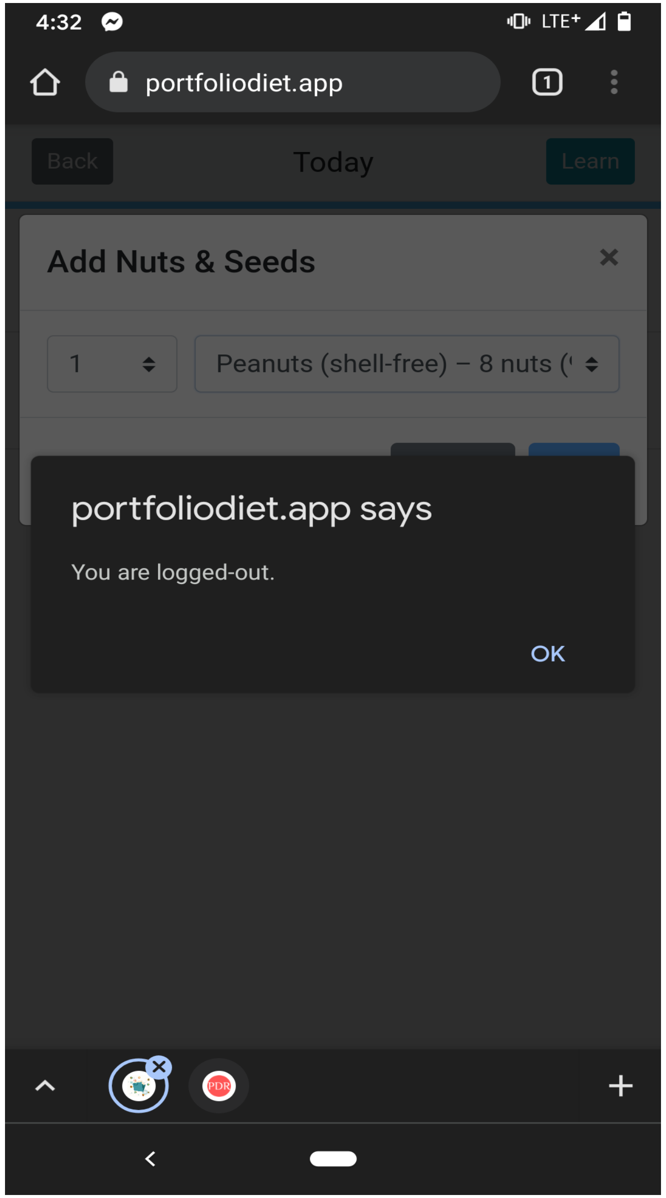

Addition of a logout popup: After 21 hours of application inactivity, the application automatically logs out the user and a pop-up window will notify the user that they have been logged off.

This is a Multimedia Appendix to a full manuscript published in the J Med Internet Res.

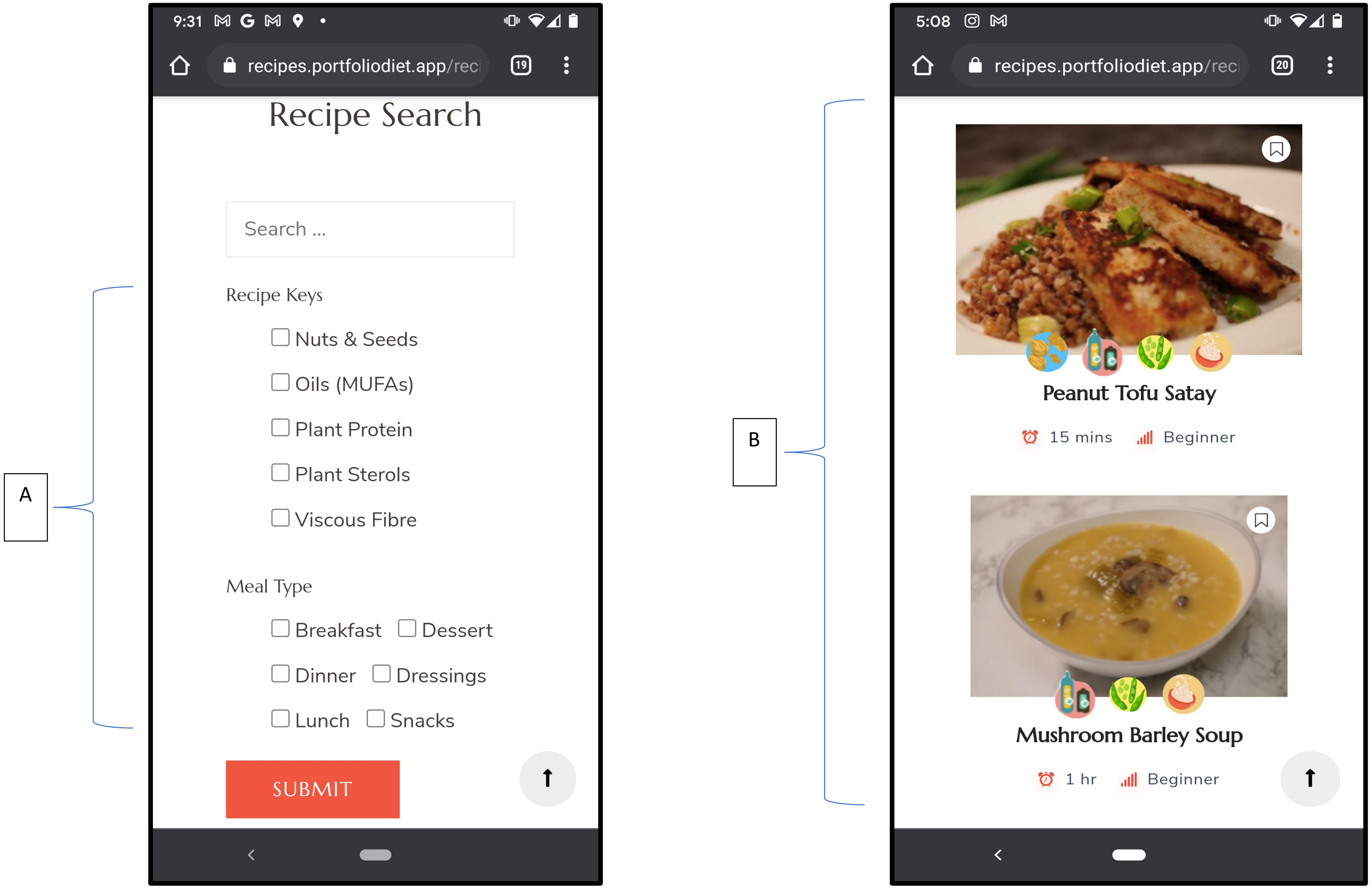

Creation of filterable recipe page: A, example of filters recipe; B, example of pages to enhance users experience of the recipes

This is a Multimedia Appendix to a full manuscript published in the J Med Internet Res.
